# A Highly Sensitive GFP Activation Assay for Detection of DNA Cleavage in Cells

**DOI:** 10.3389/fcell.2021.771248

**Published:** 2021-11-11

**Authors:** Ziying Hu, Chengdong Zhang, Daqi Wang, Siqi Gao, Sang-Ging Ong, Yongming Wang, Wei V. Zheng

**Affiliations:** ^1^ State Key Laboratory of Genetic Engineering, School of Life Sciences, Zhongshan Hospital, Fudan University, Shanghai, China; ^2^ Centre for Assisted Reproduction, Shanghai First Maternity and Infant Hospital, Tongji University School of Medicine, Shanghai, China; ^3^ Department of Pharmacology, University of Illinois College of Medicine, Chicago, IL, United States; ^4^ Division of Cardiology, Department of Medicine, University of Illinois College of Medicine, Chicago, IL, United States; ^5^ Shanghai Engineering Research Center of Industrial Microorganisms, Shanghai, China; ^6^ Intervention and Cell Therapy Center, Peking University Shenzhen Hospital, Shenzhen, China

**Keywords:** CRISPR, genome editing, DNA cleavage, off-target, PAM

## Abstract

CRISPR/Cas9 nucleases hold great potential for gene therapy, but they frequently induce unwanted off-target cleavage. We previously developed a GFP activation assay for detection of DNA cleavage in cells. Here, we demonstrate two novel applications of this assay. First, we use this assay to confirm off-target cleavage that cannot be detected by targeted deep sequencing in cells before. Second, we use this approach to detect multiple alternative PAMs recognized by SpCas9. These noncanonical PAMs are associated with low cleavage activity, but targets associated with these PAMs must be considered as potential off-target sites. Taken together, the GFP activation assay is a powerful platform for DNA cleavage detection in cells.

## Introduction

The RNA-guided CRISPR/Cas9 system can introduce desired mutations into the genome and therefore has a broad range of research and medical applications (Cong et al., 2013; Hwang et al., 2013; Mali et al., 2013; Xie et al., 2017; Wang et al., 2019a). This system consists of a Cas9 nuclease and a guide RNA (gRNA), which forms a Cas9-gRNA complex, recognizing a target sequence (protospacer) with a downstream protospacer adjacent motif (PAM), and induces a site-specific double-strand break (DSB) (Jinek et al., 2012; Cong et al., 2013; Mali et al., 2013). DSBs are repaired by either non-homologous end joining (NHEJ) or homologous recombination (HR) repair pathway, resulting in desired mutations (Wang et al., 2012; Ran et al., 2013a; Wang et al., 2014). Although the targeting specificity of Cas9 nucleases is controlled by the 20-nt guide sequence of the gRNA and the presence of a PAM adjacent to the target sequence in the genome, potential off-target cleavage could still occur (Fu et al., 2013; Hsu et al., 2013; Ran et al., 2013b; Fu et al., 2014). Off-target cleavage requires a DNA sequence with certain degrees of homology to the target sequence, followed by a PAM or a noncanonical PAM (Hsu et al., 2013; Lin et al., 2014; Wang et al., 2019a). Off-target mutations can confound interpretation of the experiments and can have implications for the development of therapeutic applications.

The importance of the off-target issue has spurred the development of multiple approaches to identify the frequencies and locations of unintended off-target mutations (Tsai and Joung, 2016). The initial approaches are *in silico* prediction of potential off-target cleavage sites based on similarity to the intended target site followed by the targeted experimental assessment of indel mutations at those locations (Fu et al., 2013; Hsu et al., 2013). Subsequently, several genome-wide unbiased approaches have been developed, including cell-based approaches (such as GUIDE-seq and HTGTS) (Crosetto et al., 2013; Frock et al., 2015; Tsai et al., 2015; Wang et al., 2015) and cell-free approaches (such as CIRCLE-seq) (Kim et al., 2015; Tsai et al., 2017). CIRCLE-seq is supposed to be the most sensitive approach, enabling the identification of rare off-target cleavage events *in vitro* (Tsai et al., 2017). However, it is a challenge to test whether the rare off-target cleavage events occur in cells. Targeted amplicon sequencing is routinely used to validate off-target mutations, but the error rate of next-generation sequencing places a floor for indel mutation detection of ∼0.1% (Tsai et al., 2017). Therefore, easy and sensitive approaches for DNA cleavage detection in cells are missing.

Previously, we developed a GFP activation assay for CRISPR PAM screening (Hu et al., 2020). In this approach, a target sequence is inserted between ATG and GFP coding sequence, disrupting GFP expression. If Cas9 nucleases cleave the target sequence and induce in-frame mutations, GFP expression will be restored. In this study, we test whether this approach can be used to detect off-target cleavage. We first demonstrate that this approach enables us to verify rare off-target cleavage events that could not be detected in cells before. Next, we demonstrate that this approach enables the detection of multiple alternative SpCas9 PAMs associated with very low cleavage activity. Therefore, this GFP activation assay is a sensitive platform for DNA cleavage detection in cells.

## Results

### The GFP Activation Assay for Validation of Off-Targets

**FIGURE 1:**
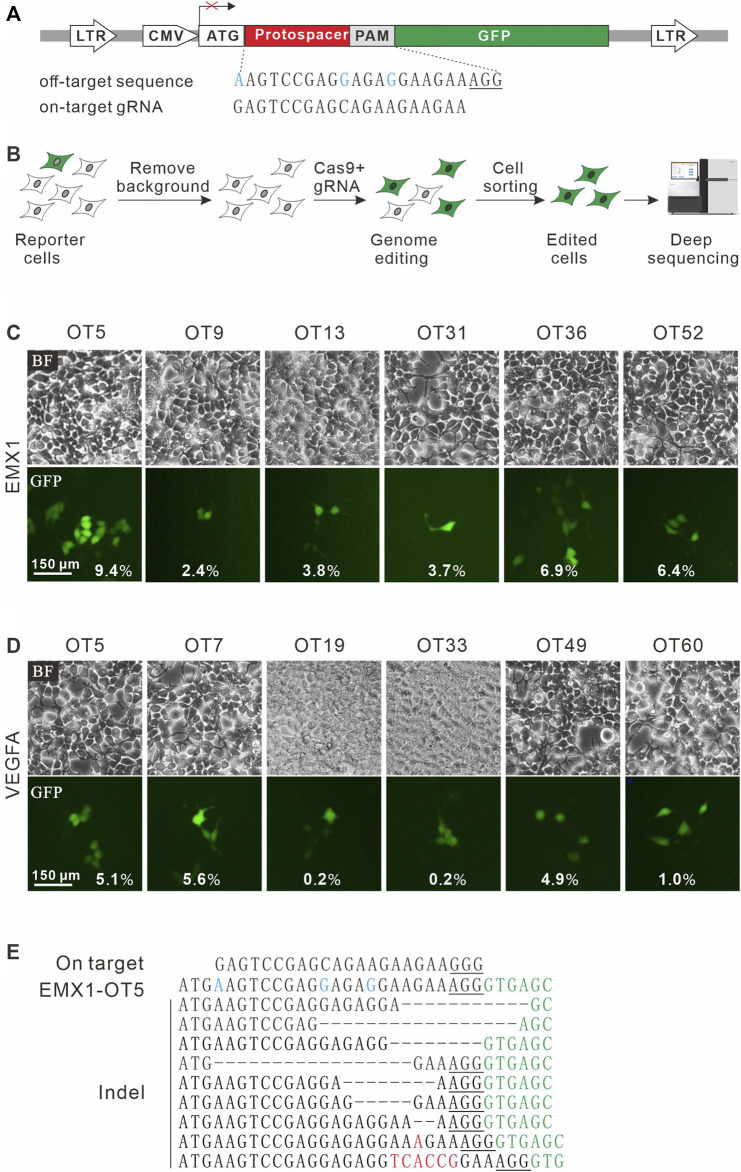
A GFP activation assay for DNA cleavage detection. **(A)** Schematic diagram of the GFP activation assay. A lentiviral vector contains a CMV-driven GFP. A protospacer followed by a PAM is inserted between ATG and GFP coding sequence, disrupting GFP expression. An example of off-target sequence as well as on-target gRNA is shown below. **(B)** The vector is stably integrated into HEK293T cells. Background GFP-positive cells are removed by cell sorting. After genome editing, a portion of cells will restore GFP expression. GFP-positive cells are sorted out and PAM sequences were PCR-amplified for deep-sequencing analysis. **(C)** Six off-target sites (for a gRNA targeting *EMX1*) identified by CIRCLE-seq are selected for validation in cells by the GFP activation assay. **(D)** Six off-target sites (for a gRNA targeting *VEGFA* site 1) identified by CIRCLE-seq are selected for validation in cells by the GFP activation assay. **(E)** Indel sequences are detected by deep sequencing for EMX1-OT5. On target sequence is shown above. Mismatches in the off-target sequence (EMX1-OT5) are shown in blue. The insertions are shown in red. The GFP sequence is shown in green. PAM is underlined.

To establish a highly sensitive approach for rare off-target cleavage detection in cells, a previous GFP activation assay was used (Hu et al., 2020). A target sequence (protospacer) with a PAM is inserted between ATG start codon and GFP coding sequence, disrupting GFP expression by frameshift mutation. When Cas9 nucleases cleave the protospacer and generate small insertions/deletions (indels), a portion of cells will restore the GFP reading frame, leading to GFP expression (Figure 1A). The reporter system was delivered into HEK293T cells by lentiviral infection to generate a stable cell line. Background GFP-positive cells caused by plasmid mutations were removed by cell sorting (Figure 1B).

CIRCLE-seq is the most sensitive approach for genome-wide off-target detection to date (Tsai et al., 2017). Tsai et al. identified up to hundreds of off-target sites for a given target by CIRCLE-seq, but they failed to validate a list of off-target sites in cells by using targeted amplicon sequencing (Tsai et al., 2017). It possible that off-target cleavage occurred at these sites but the detection assay was not sensitive enough. To test the power of the GFP activation assay, we first chose two off-target sites (EMX1-OT5 and VEGFA-OT7; Table 1) that can be detected by both CIRCLE-seq and GUIDE-seq (Tsai et al., 2017). GFP-positive cells were observed 2 days after the transfection of SpCas9 with on-target gRNA but not SpCas9 alone (Figures 1C,D; Supplementary Figures S1a,S1b). The GFP-positive cells were sorted out, and the protospacer sequences were PCR-amplified for deep sequencing. Deep sequencing analysis revealed that indels occurred (Figure 1E). These data demonstrated that the GFP activation assay enabled off-target cleavage detection in cells.

**TABLE 1:** Off-target sites of *EMX1* and *VEGFA* site 1 in this study.

Sites	Sequence^a^	Locus
EMX1-On	GAG​TCC​GAG​CAG​AAG​AAG​AAG​GG	chr2:72933853–72933875
EMX1-OT5	AAG​TCC​GAG​GAG​AGG​AAG​AAA​GG	chr1:23384119–23394141
EMX1-OT9	GAG​TAC​AAG​CAG​ATG​AAA​AAC​GG	chr10:126391610–126391632
EMX1-OT13	GAG​GCC​AAG​CAG​AAA​GAA​AAA​GG	chr7:31861458–31861480
EMX1-OT31	GAC​TCC​GAG​CAG​CAG​AAG​GAT​GG	chr2:65555376–65555398
EMX1-OT36	GAG​TTA​GAG​CAG​AGG​AAG​AGA​GG	chr6:99251280–99251302
EMX1-OT52	GTG​TCA​GAG​CAG​AAA​AAG​AGT​GG	chr4:25059119–25059141
VEGFA-On	GGG​TGG​GGG​GAG​TTT​GCT​CCT​GG	chr6:43769554–43769576
VEGFA-OT5	GGG​GGC​AGG​GAG​ATT​GCT​CCT​GG	chr18:366708–366730
VEGFA-OT7	AAG​TAA​GGG​AAG​TTT​GCT​CCT​GG	chr16:869350–869372
VEGFA-OT19	GGG​AGG​AGA​GAG​TTT​GCT​CTC​TG	chr8:139702078–139702100
VEGFA-OT33	AGA​GGG​GTG​GAG​TTT​GTT​CCA​GG	chr20:38342497–38342519
VEGFA-OT49	GGG​GAG​GGG​GAG​ATG​GCT​CCC​GG	chr10:113530631–113530653
VEGFA-OT60	GAG​GTG​GGG​TGA​TTT​GCT​CCA​GG	chr11:57067754–57067776

a PAM sequences were shown in green and mismatched nucleotides were shown in red.

We chose 10 additional off-target sites (five for *EMX1* and five for *VEGFA* site 1; Table 1) that were detected by CIRCLE-seq but failed to be validated by targeted amplicon sequencing (Tsai et al., 2017). GFP-positive cells were observed for all of the tested sites 2 days after the transfection of SpCas9 with on-target gRNA but not SpCas9 alone (Figures 1C,D; Supplementary Figures S1a,S1b), indicating that off-target mutations could occur with these sites in cells. Indels were confirmed by deep sequencing (Supplementary Figures S2, S3). These data demonstrated that the GFP activation assay was a highly sensitive platform for DNA cleavage detection.

### The GFP Activation Assay for Detection of New Non-Canonical PAMs of SpCas9

**FIGURE 2:**
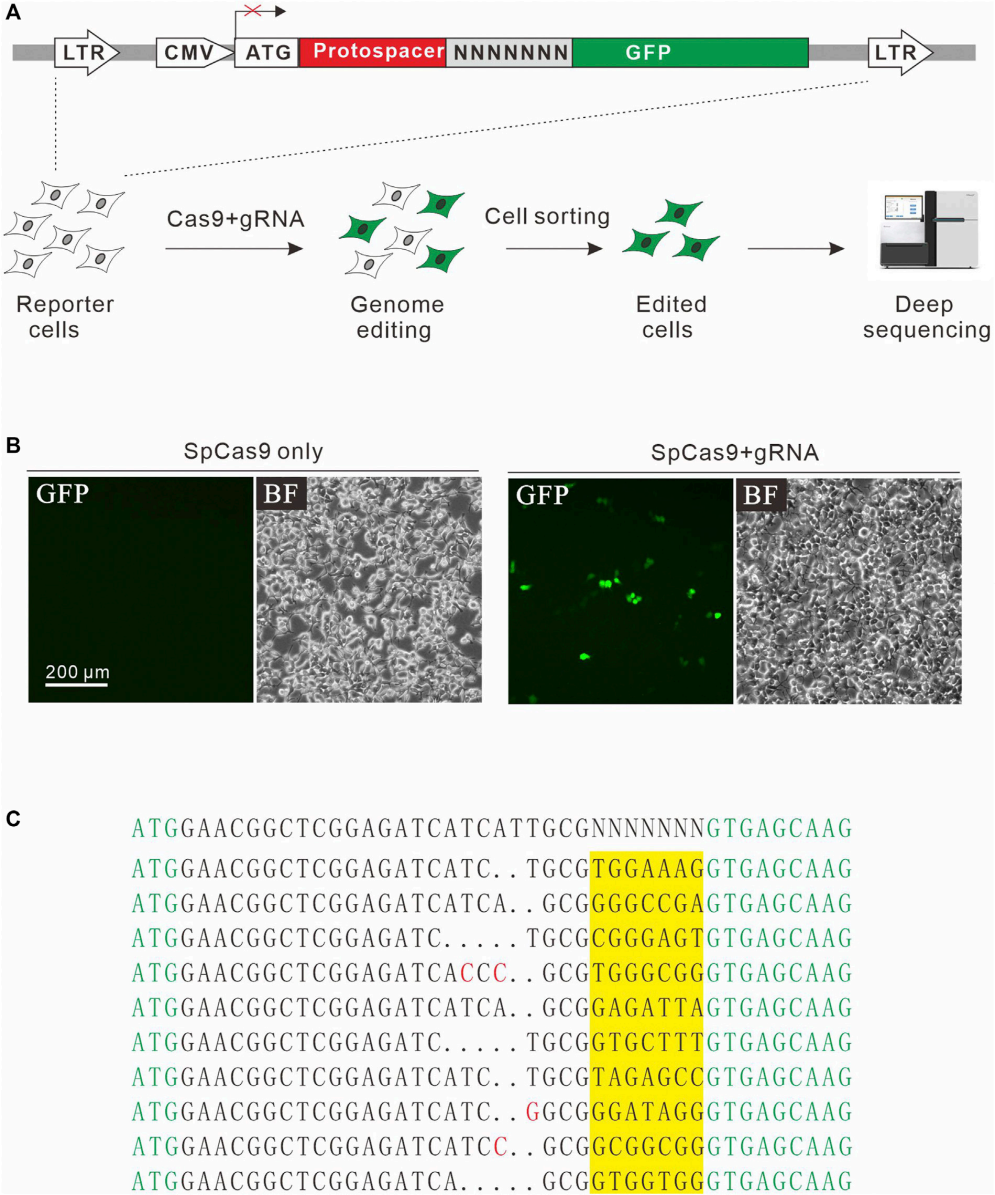
A GFP activation assay for PAM screening. **(A)** Schematic diagram of the GFP activation assay. A lentiviral vector contains a CMV-driven GFP. A target sequence followed by a 7-bp random sequence is inserted between ATG and GFP coding sequence, disrupting GFP expression. The library is stably integrated into HEK293T cells. After genome editing, a portion of cells will restore GFP expression. GFP-positive cells are sorted out and PAM sequences were PCR-amplified for deep-sequencing analysis. **(B)** Transfection of *Sp*Cas9 and gRNA results in GFP expression, while transfection of *Sp*Cas9 alone cannot induce GFP expression. **(C)** Deep-sequencing reveals that targets with multiple PAMs can be edited. GFP sequence is shown in green; insertion mutations are shown in red; 7-bp random sequences are a highlight in yellow.

In addition to NGG PAM, SpCas9 also recognizes noncanonical PAMs, including NAG, NCG, NGA, and NNGG (Jiang et al., 2013; Doench et al., 2016). We investigate whether the GFP activation assay is sensitive enough to identify additional PAMs. For this purpose, a protospacer containing 7nt downstream randomized DNA sequences was inserted between ATG start codon and GFP coding sequence, disrupting GFP expression (Figure 2A). The GFP reporter construct was stably inserted into the genome. Quantitative PCR (qPCR) revealed that there were average 3.82 copies of construct per cells. GFP-positive cells could be observed 2 days after the transfection of SpCas9 with the corresponding gRNA but not SpCas9 alone (Figure 2B). The GFP-positive cells were sorted out, and the protospacer with randomized DNA sequences were PCR-amplified for deep sequencing. Deep sequencing analysis revealed that indels associated with different PAMs could be detected (Figure 2C).

**FIGURE 3:**
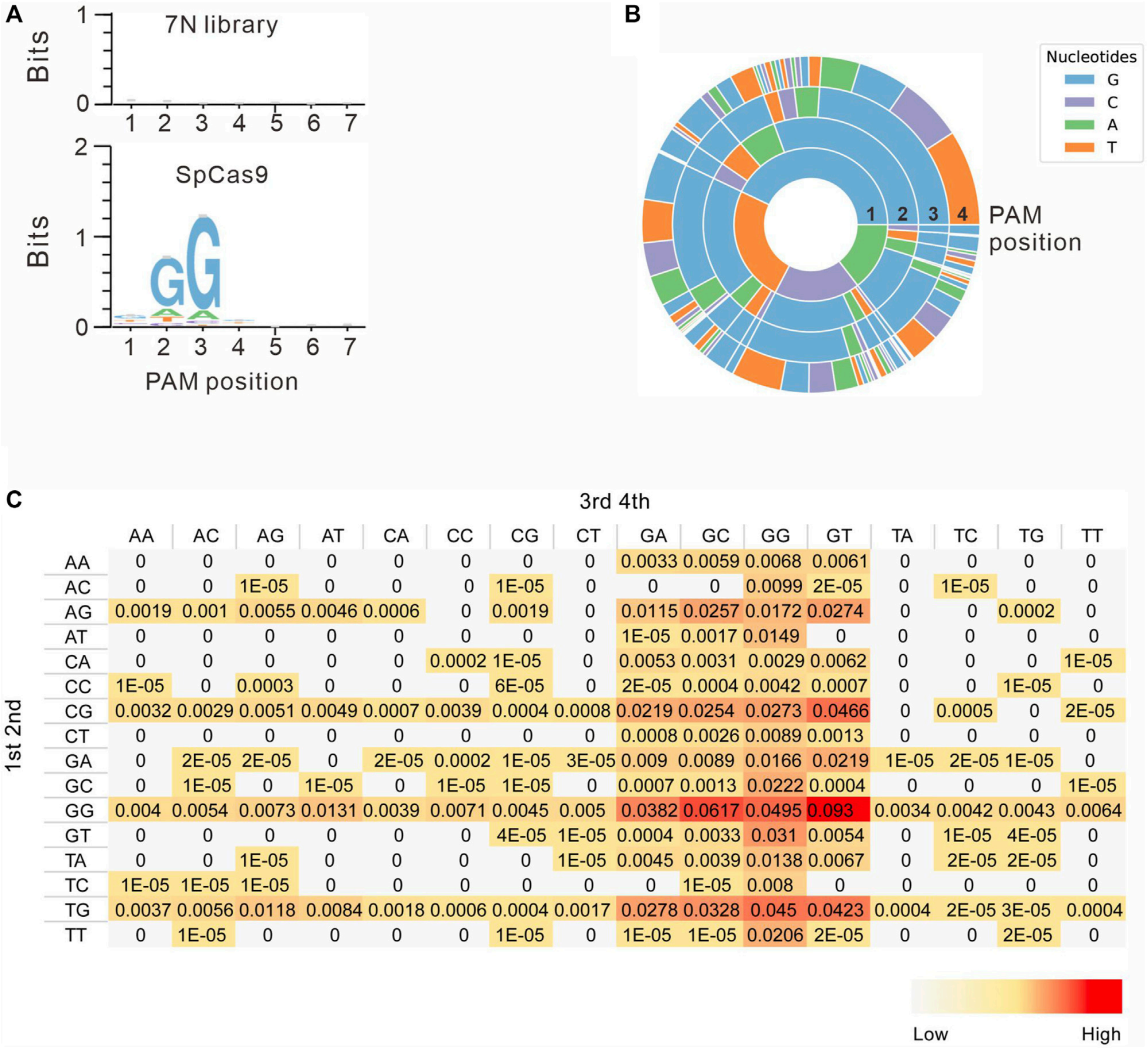
PAM sequence analysis. **(A)** WebLogo is generated based on the PAM screening assay. The upper panel is the control cells without genome editing. the lower panel is the cells after genome editing. **(B)** PAM wheel is generated based on the PAM screening assay. **(C)** Heat map for efficiency ratio of top all 256 PAMs. The 1–2 PAM sequences are shown on the left, and the 3–4 PAM sequences are shown on the top.

Next, we systematically analyzed the PAM sequences recognized by SpCas9 from deep sequencing data. Only in-frame mutations were considered as novel mutations induced by SpCas9, thus minimizing the background mutations derived from library construction or deep sequencing. Since indels could disrupt the randomized DNA sequences, GCG triple-nucleotide was used to fix the 7nt randomized DNA sequences (Figure 2C). Both WebLogo and PAM wheel captured the canonical NGG as the most enriched PAM, but the sequences other than NGG were also observed (Figures 3A,B). The nucleotide preference at position 1–4 was not random. We analyzed the PAM frequencies for all possible NNNN PAM sequences (Figure 3C). The top 74 PAMs (frequency over 0.1%) included all NNGG, NAGN, NGAN, and GGYN PAMs (Supplementary Figure S4). The most efficient PAM was NGGN, as expected, followed by NNGG, NAGN, NGAN, and GGYN (Figure 3C). Interestingly, some noncanonical PAMs such as GTGG, GCGG, and GAGT displayed comparable efficiency to NGGN PAMs.

**FIGURE 4:**
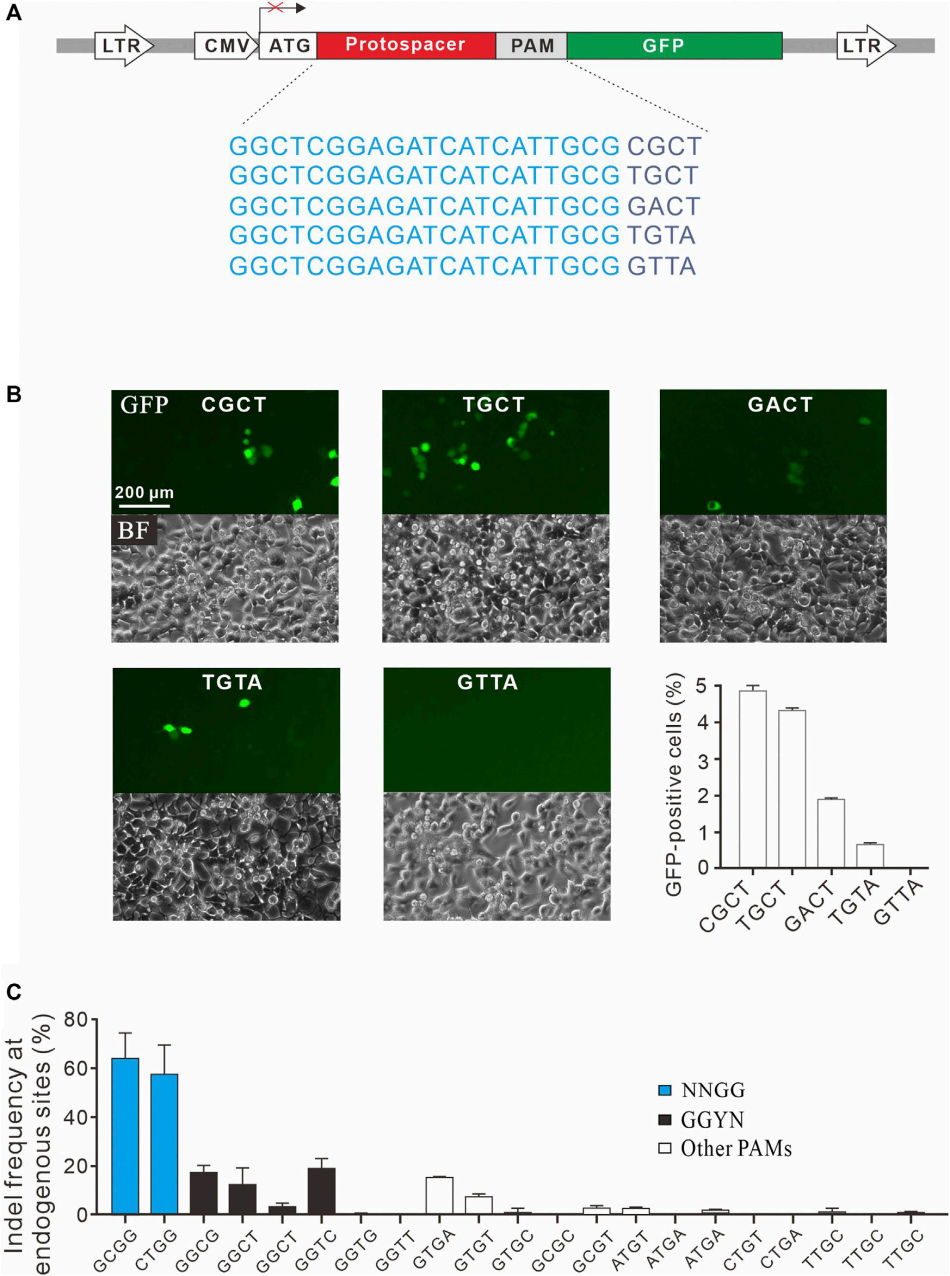
Validation of noncanonical PAMs. **(A)** Schematic diagram of the GFP activation assay with five targets shown below. **(B)** Five GFP reporter constructs with noncanonical PAMs are isolated from the PAM library and tested for genome editing. Quantification is shown on the bottom right. *n* = 3. **(C)** Indel frequency of endogenous target sequences associated with noncanonical PAMs. *n* = 3.

To confirm novel PAMs identified here, we isolated four GFP reporter constructs with different PAM sequences (CGCT, TGCT, GACT, and TGTA) from the PAM library and established stable cell lines for each construct (Figure 4A). Transfection of SpCas9 with gRNA induced GFP expression for all of them, indicating that cleavage occurred with these PAMs (Figure 4B). We also isolated a construct with GTTA PAM as a negative control. Consistent with the PAM screening results, GFP-positive cells could not be observed with GTTA PAM (Figure 4B). We further tested 21 endogenous targets with noncanonical PAMs. Two NNGG PAMs displayed very high activity, with indel rates of 64.3 and 57.6% for GCGG and CTGG, respectively (Figure 4C). Three GGYN PAMs (GGCG, GGCT, and GGTC) as well as GTGA and GTGT PAMs also displayed significant activity. Other PAMs only displayed minimal activity. These novel PAMs are useful for *in silico* prediction of potential off-target sites.

## Discussion

In this study, we demonstrate that the GFP activation assay is a highly sensitive platform for DNA cleavage detection in cells. Targeted amplicon sequencing is routinely used to detect DNA cleavage, but the error rate of next-generation sequencing places a floor for indel mutation detection of ∼0.1% (Tsai et al., 2017). Our GFP activation assay requires a cell sorting step to enrich the cleavage events before targeted amplicon sequencing, which increases the sensitivity. GFP-disruption assay can be used for detection of DNA cleavage (Permyakova et al., 2019), but it cannot detect the rare cleavage events due to the background of GFP-negative cells. In addition, GFP-disruption assay does not allow us to test non-GFP DNA sequence. “Traffic light” system is an elegant design for detection of DNA cleavage in cells (Certo et al., 2011). This system contains a GFP cassette disrupted by a target sequence and an RFP cassette which is out of frame. If DNA cleavage occurs and DSB is repaired by NHEJ, the RFP expression will be activated. The sensitivity of this system should be comparable to our assay.

In the GFP activation assay, the GFP reporter construct is inserted into the genome by lentiviruses, which preferentially integrate into transcriptionally active regions (Schröder et al., 2002; Mitchell et al., 2004; Kim et al., 2017). These regions are much more accessible for the CRISPR/Cas9 machinery. One limitation of this assay is that it may not reflect the real situation in the therapeutically relevant cells, where endogenous off-target sites may be not accessible for Cas9 nucleases. On the other hand, GFP activation assay has an advantage over detection at endogenous loci. When Cas9+gRNA are delivered into the human body by adeno-associated virus (AAV) (Wang et al., 2019b), AAV can also infect therapeutically irrelevant cell types, where the off-target sites may be open. It is not safe enough to only consider therapeutically relevant cells.

The GFP activation assay requires to clone every potential off-target site into the vector and establishes stable cell lines, which is time-consuming. For clinical use with CRISPR/Cas9, it deserves to test very rare cleavage events happened at off-targets. It is also possible to synthesize all potential off-targets with microarray and clone them into the vector to establish a library. The library is stably integrated into chromosome. Transfection of Cas9+gRNA can induce GFP expression. GFP-positive cells are sorted out and synthesized off-targets are PCR-amplified for deep sequencing. This procedure may allow high-throughput test of potential off-targets with GFP activation assay.

SpCas9 is the most extensively studied and applied system to date due to its high efficiency and simple PAM requirement (Cong et al., 2013; Mali et al., 2013). In addition to NGG PAM, several noncanonical PAMs including NAG, NCG, NGA, and NNGG have been identified (Jiang et al., 2013; Doench et al., 2016). These noncanonical PAMs are associated with low cleavage activity, but targets associated with them must be considered as potential off-target sites. Therefore, it is crucial to identify additional noncanonical PAMs for SpCas9. The GFP activation assay enables to identify multiple noncanonical PAMs, dramatically extending the list of noncanonical SpCas9 PAMs. We anticipate that the GFP activation assay is also useful for other applications that require highly sensitive DNA cleavage detection, such as the test of new genome tools.

## Methods

### Cell Culture and Transfection

HEK293T cells were maintained in Dulbecco’s Modified Eagle Medium (DMEM) supplemented with 10% FBS (Gibco) and 1% antibiotics at 37°C with 5% CO_2_. For the PAM library screen, HEK293T cells were plated into 10 cm dishes, and transfected at ∼60% confluency with Cas9-gRNA-expressing plasmid (15 μg) using Lipofectamine 2000 (Life Technologies). For PAM validation at endogenous sites, HEK293T cells were seeded on 48-well plates and transfected with Cas9-gRNA-expressing plasmid (500 ng) using Lipofectamine 2000 (Life Technologies).

### The PAM Library Construction

The DNA oligonucleotides containing a target sequence followed by random sequences and flanking homologous sequences (for Gibson Assembly) were synthesized from GENEWIZ (Suzhou, China). Full-length oligonucleotides were PCR-amplified using Q5 High-Fidelity 2X Master Mix (NEB), size-selected using a 3% agarose gel EX (Life Technologies, Qiagen), and purified using MinElute Gel Extraction Kit (Qiagen). PCR products were cloned into a lentiviral vector by Gibson Assembly (NEB) and purified with Agencourt AMPure XP SPRI beads (Beckman Coulter). The Gibson Assembly products were electroporated into MegaX DH10B^™^ T1^R^ Electrocomp^™^ Cells (Invitrogen) using a GenePulser (BioRad). The bacteria were added into recovery media and grew at 32°C, 225 rpm for 14 h. The plasmid DNA was extracted from bacteria using Endotoxin-Free Plasmid Maxiprep (Qiagen). The plasmid sequence is shown in Supplementary Figure S5. All primers and gRNA sequences used in this study were listed in Supplementary Table S1.

### Lentivirus Production

For PAM library packaging, HEK293T cells were seeded in three 10 cm dishes and transfected at ∼40% confluency. For each dish, 12 μg of PAM library plasmid, 9 μg of psPAX2, and 3 μg of pMD2.G were transfected with 60 μL of Lipofectamine 2000 (Life Technologies). Viruses were harvested twice at 48 and 72 h post-transfection. The viruses were concentrated using PEG8000 (no. LV810A-1, SBI, Palo Alto, CA), dissolved in PBS and stored at −80°C. For single PAM reporter construct packaging, HEK293T cells were seeded into 6-well plates, 1.2 μg of PAM reporter plasmid, 0.9 μg of psPAX2, and 0.3 μg of pMD2.G were transfected with 5 μL of Lipofectamine 2000. Viruses were harvested twice at 48 and 72 h post-transfection.

### PAM Library Screening Assay

HEK293T cells were plated into a 15 cm dish at ∼30% confluence. After 24 h, cells were infected with PAM library lentiviruses with at least 1000-fold coverage of each PAM. 24 h after infection, the cells were selected with 2 µg/ml of puromycin for 5 days. To remove plasmid mutations that induce GFP expression, the GFP negative cells were sorted out with a MoFlo XDP machine (Beckman Coulter) and seeded into 10 cm dishes. The residual GFP-positive cells were removed pipette tips under microscope. For the PAM screen, PAM library cells were transfected with Cas9-gRNA expressing plasmid for 3 days. The GFP-positive cells were sorted out by MoFlo XDP machine and the genomic DNA was isolated using TIANamp Genomic DNA Kit (TIANGEN) following the manufacturer’s instructions. DNA target sites were PCR-amplified by nested PCR with Q5 High-Fidelity 2X Master Mix (NEB). First, the target region was PCR-amplified using primers Deep-F1/R1 with 25 cycles; second, 3 μL of PCR products from the first step were used as a template and amplified by primers P5-adapter-F and P7-adapter-R for 15 cycles. The PCR products were purified using the Gel Extraction Kit (Qiagen) and sequenced on Illumina HiSeq X by 150-bp paired-end sequencing.

### PAM Sequence Analysis

Twenty base-pair sequences (AAG​CCT​TGT​TTG​CCA​CCA​TG/GTG​AGC​AAG​GGC​GAG​GAG​CT) flanking the target sequence (GAACGGCTCGGAGATCATCATTGCGNNNNNNN) were used to fix the target sequence. Only target sequences with in-frame mutations were used for PAM analysis. GCG and GTG​AGC​AAG​GGC​GAG​GAG​CT sequences were used to fix a 7-bp random sequence. Only intact 7-bp random sequences were used for PAM analysis. The 7-bp random sequences were extracted and visualized by WebLog3 (Crooks et al., 2004) and wheel chart (Leenay et al., 2016) to demonstrate PAMs.

### Plasmid Constructs and DNA Cleavage Detection

Plasmids containing specific PAM sequences were isolated from the PAM library. Plasmids of EMX1-OT and VEGFA-OT construction: vector backbone of the plasmid containing specific PAM sequences was PCR-amplified using primers EMX1/VEGFA-OT-F/cozak-R, followed by phosphorylation with T4 Polynucleotide Kinase (NEB) and religation with T4 DNA ligase (NEB). Each plasmid was packed into lentiviruses to generate a stable cell line. To remove plasmid mutations that induce GFP expression, the GFP-negative cells were sorted out by the MoFlo XDP machine (Beckman Coulter). The cells were seeded into 24-well and transfected with 800 ng of SpCas9-gRNA-expressing plasmid (PX459, addgene #118632) by Lipofectamine 2000 (Life Technologies). Five days after transfection, the GFP-positive cells were measured on the Calibur instrument (BD). Data were analyzed using FlowJo. Besides, GFP-positive cells of EMX1-OT, and VEGFA-OT were sorted out by the MoFlo XDP machine (Beckman Coulter). The genomic DNA was isolated and target sites were amplified by nested PCR and extracted by Gel Extraction Kit (QIAGEN). The amplicons are prepared for deep sequencing.

### Test of PAM Activity at Endogenous Sites

HEK293T cells were seeded on 24-well plates and transfected with epiCRISPR(4) constructs expressing SpCas9 and gRNA followed by puromycin selection (2 µg/ml) for 6 days. The genomic DNA was isolated, and the target sites were PCR-amplified by nested PCR for deep sequencing.

### Quantification of GFP Reporter Copy Number

The genomic DNA was isolated from GFP activation cells using TIANamp Genomic DNA Kit (TIANGEN) following the manufacturer’s instructions. To generate standard curve, GFP reporter plasmid was serially diluted to final concentrations of 10^9^,10^8^,10^7^,10^6^,10^5^,10^4^,10^3^,10^2^ copies/μL. Quantitative PCR were performed with CFX Connect Real-Time System (Bio-Rad) and 2X SYBR Green qPCR Master Mix (APExBIO). Use the following parameters: 1 cycle at 95°C for 2 min, followed by 40 cycles of 95°C for 15 s, 55°C for 30 s, and 72°C for 30 s. Each sample was quantified in triplicate. The same parameters were used to quantify GFP copy numbers in the genomic DNA. Absolute transgene copy numbers were calculated with C_q_ values and dilution factor based on standard curve.

### Quantification and Statistical Analysis

All the data are shown as the mean ± S.D. Statistical analyses were conducted using Microsoft Excel.

## Data Availability

The data presented in this study are deposited in the NCBI SRA BioProject repository, accession number is PRJNA773240.
