# Supplementary material for: A Highly Sensitive GFP Activation Assay for Detection of DNA Cleavage in Cells
Source: Front Cell Dev Biol. 2021 Nov 11;9:771248. doi: 10.3389/fcell.2021.771248 (PMC8636026; doi:10.3389/fcell.2021.771248)
Supplement: Supplementary file 1 [file DataSheet1.PDF]

A GFP activation assay for highly sensitive detection of off-target cleavage

Ziying Hu, Chengdong Zhang, Daqi Wang, Siqi Gao, Yongming Wang, Wei V. Zheng

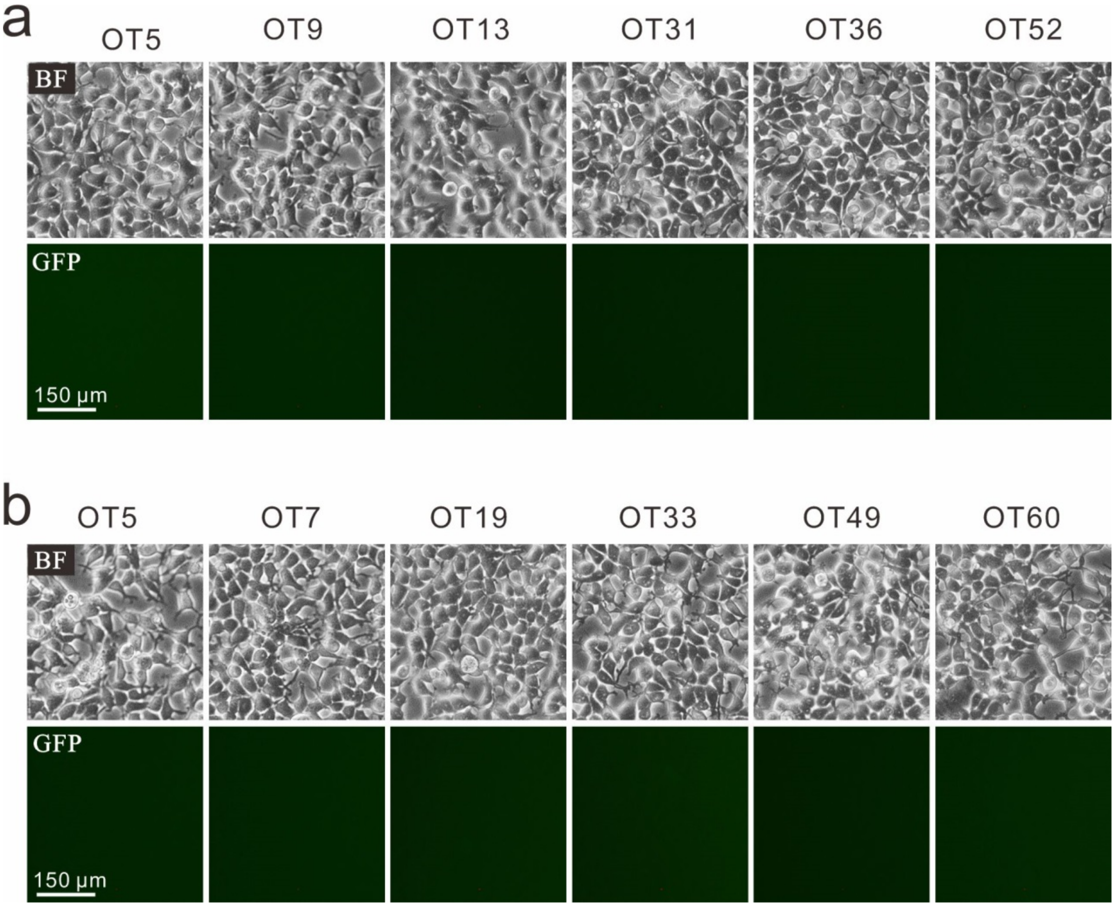

Supplementary Figure 1. Negative controls for the GFP activation assay. a, Negative controls for *EMX1* off-targets. Only SpCas9 expressing vector is transfected into the cells. **b,** Negative controls for *VEGFA* site 1 off-targets. Only SpCas9 expressing vector is transfected into the cells.

EMX1-OT9-Indel

```
ATGGAGTACAAGCAGATGAAAAACGGGTGAGCAAG
ATGGAGTACAA-----------ACGGGTGAGCAAG
ATGAAGTCCGAG-----GAAA---GGGTGAGCAAG
ATGGAGTACAAGCAGATGAA-----GGTGAGCAAG
ATGGAGTACAAGCAGATGAA—ACGGGTGAGCAAG
ATGGAGTACAAGCAGATGGA--ACGGGTGAGCAAG
ATGGAGTACAAGC-----------GGGTGAGCAAG
ATGGAGTACAAGCAGATGA-----------GCAAG
ATGGAGTACAAGCAG-----------GTGAGCAAG
ATGGAGTACAAGCAGATGAAAAAACGGGTGAGCAAG
ATGGAGTACAAGCAGATGAGCAAGTACAAACGGGTGAGCAAG
```

EMX1-OT13-Indel

```
ATGGAGGCCAAGCAGAAAGAAAAAGGGTGAGCAAG
ATGGAGGCCAAGCAGAAAGAAAAAAGGGTGAGCAAG
ATGGA-----------------AAGGGTGAGCAAG
ATGGAGGCCAAGCAGAAAGA--AAGGGTGAGCAAG
ATGGAGGCCAAGCAGAAA--AAAAGGGTGAGCAAG
ATGGAGGCCAAGCAGAA-G-AAAAGGGTGAGCAAG
ATGGACTCCGAGCAG-CAGA-GATGGGTGAGCAAG
ATGGAGGCCAAGCAGAAA-----AGGGTGAGCAAG
ATGGAGGCCAAGCAGAAAGA-----GGTGAGCAAG
ATGGAGGCCATGGAG-----------GTGAGCAAG
ATGGAGGCTTTGCCT-----------GTGAGCAAG
```

EMX1-OT31-Indel

```
ATGGACTCCGAGCAGCAGAAGGATGGGTGAGCAAG
ATGG-----------------GATGGGTGAGCAAG
ATGGAC-CCT-------------TGGGTGAGCAAG
ATGGACTCCGAGCAGCAGA-----------GCAAG
ATGGACTCCGAGCAGCAGAA-----------CAAG
ATGGACTCCGAGCAGCAG--------GTGAGCAAG
ATGGACTCCGAGCAGCAGA--------TGAGCAAG
ATGGACTCCGAGCAGCAGAT-----GGTGAGCAAG
ATGGACTCCGAGCAGCAGAA--ATGGGTGAGCAAG
ATGGACTCCGAGCAGCAGA--GATGGGTGAGCAAG
ATGGACTCCGAGCAGCAGAAAGGATGGGTGAGCAAG
```

EMX1-OT36-Indel

```
ATGGAGTTAGAGCAGAGGAAGAGAGGGTGAGCAAG
ATGGAGTTAGAGCAGAGGAAAGAGAGGGTGAGCAAG
ATGGAG-----------------AGGGTGAGCAAG
ATGGAGTTAGAGCAGAG--------------CAAG
ATGGAGTTAGAG-----------AGGGTGAGCAAG
ATGGAGTTAGAGCAGAGGA-----------GCAAG
ATGGAGTTAGAGCAGAGG--------GTGAGCAAG
ATGGAGTTAGAGCAGAGGAG--------CAGCAAG
ATGGAGTTAGAGCAGAGGA--------TGAGCAAG
ATGGAGTTAGAGCAGAGGA-----GGGTGAGCAAG
ATGGAGTTAGAGCAGAGGAA-----GGTGAGCAAG
ATGGAGTTAGAGCAGAGGAA--GAGGGTGAGCAAG
```

EMX1-OT52-Indel

```
ATGGTGTCAGAGCAGAAAAAGAGTGGGTGAGCAAG
ATGGTGTCAGAGCAGAAAAAAGAGTGGGTGAGCAAG
ATGG-----------------AGTGGGTGAGCAAG
ATGGTGT-----------------GGGTGAGCAAG
ATGGTGTCAGAGCAGA--------------GCAAG
ATGGTGTCAGAG--------------GTGAGCAAG
ATGGTGTCAGAG-----------TGGGTGAGCAAG
ATGGTGTCAGAGCAGAAAA-----------GCAAG
ATGGTGTCAGAGCAGAA-----GTGGGTGAGCAAG
ATGGTGTCAGAGCAG--------TGGGTGAGCAAG
ATGGTGTCAGAGCAGAAA--GAGTGGGTGAGCAAG
```

Supplementary Figure 2. Indel sequences are detected by deep sequencing for *EMX1* off-targets.

VEGFA-OT5-Indel

```
ATGGGGGGCAGGGAGATTGCTCCTGGGTGAGCAAG
ATGGGGGGCAGGGAGATTGCCTCCTGGGTGAGCAAG
ATGGGGGGCAGGGAGATTGC--CTGGGTGAGCAAG
ATGGGGGG-----------------GGTGAGCAAG
ATGGGGGGCAGGGAGA--------------GCAAG
ATGGGGGGCAGGGAGAT--------------CAAG
ATGGGGGGCAGGG--------------TGAGCAAG
ATGGGGGGC--------------TGGGTGAGCAAG
ATGGGGGGCAGGGAGATTGC-----------CAAG
ATGGGGGGC-----------TCCTGGGTGAGCAAG
ATGGGGGGCAGGGAA--------TGGGTGAGCAAG
ATGGGGGGCAGGGAGA-----CCTGGGTGAGCAAG
```

VEGFA-OT7-Indel

```
ATGGTAAGTAAGGGAAGTTTGCTCCTGGGTGAGCAAG
ATGGTAAGTAAGG----------------TGAGCAAG
ATGGTAAGTAAGGGAAG----------------CAAG
ATGGTAAGTAAGGGAAGTTT-------------CAAG
ATGGTAAGTAAGGGAAGT-------------AGCAAG
ATGGTAAGTAAGGGAAGTTTG----------AGCAAG
ATGGTAAGTAAG----------TCCTGGGTGAGCAAG
ATGGTAAGTAAGGGAAGTTTG-------GTGAGCAAG
ATGGTAAGTAAGGGAAGTAAG-------GTGAGCAAG
ATGGTAAGTAAGGGAAGTTTGC----GGGTGAGCAAG
ATGGTAAGTAAGGGAAGTTA----CTGGGTGAGCAAG
ATGGTAAGTAAGGGAAGTTTGC-CCTGGGTGAGCAAG
ATGGTAAGTAAGGGAAGTTTG-TCCTGGGTGAGCAAG
```

VEGFA-OT19-Indel

```
ATGGGGAGGAGAGAGTTTGCTCTCTGGTGAGCAAG
ATGGGGAGGAGAGAGTTTGC--TCTGGTGAGCAAG
ATGGGGAGGAGAGAGTTTGCTTCTCTGGTGAGCAAG
```

VEGFA-OT33-Indel

```
ATGAGAGGGGTGGAGTTTGTTCCAGGGTGAGCAAG
ATGAGAGGGGTGGAGTTTGTTTCCAGGGTGAGCAAG
```

VEGFA-OT49-Indel

```
ATGGGGGAGGGGGAGATGGCTCCCGGGTGAGCAAG
ATGGGGGAGG-----------------TGAGCAAG
ATGGGGGAGGGGGAGA--------------GCAAG
ATGGGGGAGGGGG--------------TGAGCAAG
ATGGGGGAGGGGGAGATG-----------AGCAAG
ATGGGGGAGGGGGAGATGGC-----------CAAG
ATGGGGGAGGGGGAGATGGC--------GAGCAAG
ATGGGGGAGGGGGAGATGG--------TGAGCAAG
ATGGGGGAGGGG--------TCCCGGGTGAGCAAG
ATGGGGGAGGGGGAG-----TCCCGGGTGAGCAAG
ATGGGGGAGGGGGAGATG--TCCCGGGTGAGCAAG
ATGGGGGAGGGGGAGATGGC--CCGGGTGAGCAAG
```

VEGFA-OT60-Indel

```
ATGGAGAGGTGGGGTGATTTGCTCCAGGGTGAGCAAG
ATGGAGAGGTGGG----------------TGAGCAAG
ATGGAGAGGTGGGGT----------------AGCAAG
ATGGAGAGGT-------------CCAGGGTGAGCAAG
ATGGAGAGGTGGGGTGATTTG----------AGCAAG
ATGGAGAGGTGGGGTGATTTGC----GGGTGAGCAAG
ATGGAGAGGTGGGGTGATT----CCAGGGTGAGCAAG
ATGGAGAGGTGGGGTGATTTGC-CCAGGGTGAGCAAG
ATGGAGAGGTGGGGTGATTTG-TCCAGGGTGAGCAAG
ATGGAGAGGTGGGGTGATTTGCGCTCCAGGGTGAGCAAG
ATGGAGAGGTGGGGTGATTTGCCTTGCTCCAGGGTGAGCAAG
```

Supplementary Figure 3. Indel sequences are detected by deep sequencing for *VEGFA* site 1 off-targets.

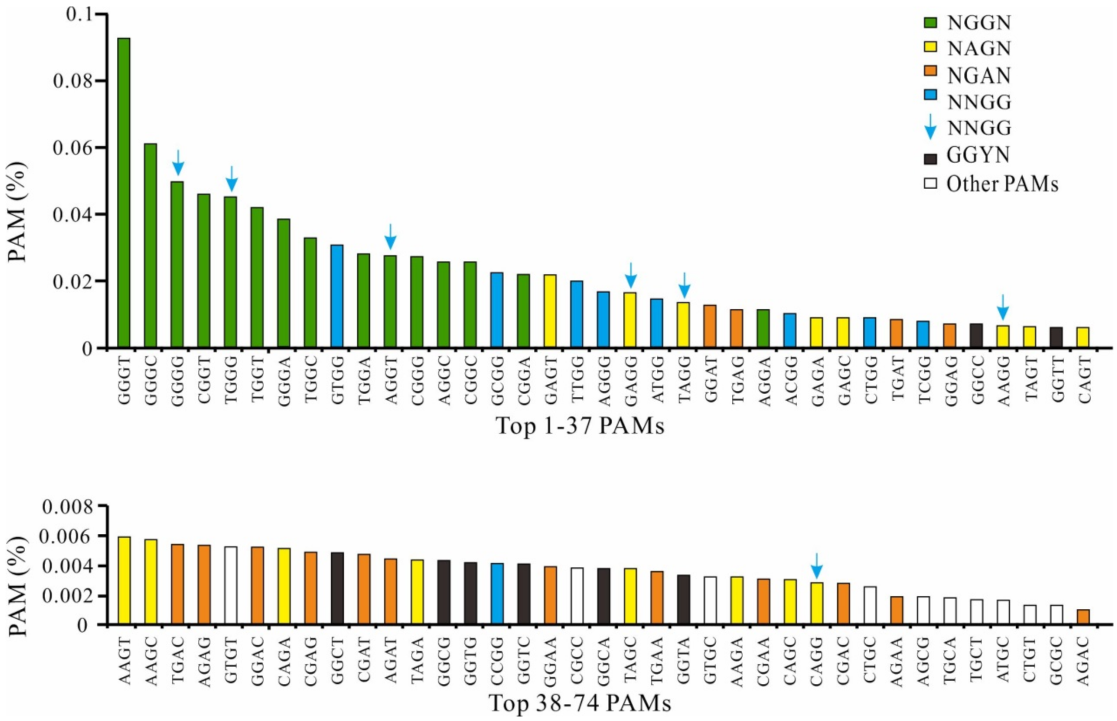

Supplementary Figure 4. PAM frequency of the top 74 PAMs. NNGG PAM overlapped with other PAMs is indicated by arrows.

ACGCGTGTAGTCTTATGCAATACTCTTGTAGTCTTGCAACATGGTAACGATG
AGTTAGCAACATGCCTTACAAGGAGAGAAAAAGCACCGTGCATGCCGATT
GGTGGAAGTAAGGTGGTACGATCGTGCCTTATTAGGAAGGCAACAGACGG
GTCTGACATGGATTGGACGAACCACTGAATTGCCGCATTGCAGAGATATTG
TATTTAAGTGCCTAGCTCGATACAATAAACGGGTCTCTCTGGTTAGACCAGA
TCTGAGCCTGGGAGCTCTCTGGCTAACTAGGGAACCCACTGCTTAAGCCTC
AATAAAGCTTGCCTTGAGTGCTTCAAGTAGTGTGTGCCCGTCTGTTGTGTG
ACTCTGGTAACTAGAGATCCCTCAGACCCTTTTAGTCAGTGTGGAAAATCT
CTAGCAGTGGCGCCCGAACAGGGACCTGAAAGCGAAAGGGAAACCAGAG
CTCTCTCGACGCAGGACTCGGCTTGCTGAAGCGCGCACGGCAAGAGGCGA
GGGGCGGCGACTGGTGAGTACGCCAAAAATTTTGACTAGCGGAGGCTAGA
AGGAGAGAGATGGGTGCGAGAGCGTCAGTATTAAGCGGGGGAGAATTAGA
TCGCGATGGGAAAAAATTCGGTTAAGGCCAGGGGGAAAGAAAAAATATAA
ATTAAAACATATAGTATGGGCAAGCAGGGAGCTAGAACGATTCGCAGTTAA
TCCTGGCCTGTTAGAAACATCAGAAGGCTGTAGACAAATACTGGGACAGCT
ACAACCATCCCTTCAGACAGGATCAGAAGAACTTAGATCATTATATAATACA
GTAGCAACCCTCTATTGTGTGCATCAAAGGATAGAGATAAAAGACACCAAG
GAAGCTTTAGACAAGATAGAGGAAGAGCAAAACAAAAGTAAGACCACCG
CACAGCAAGCGGCCACTGATCTTCAGACCTGGAGGAGGAGATATGAGGGA
CAATTGGAGAAGTGAATTATATAAATATAAAGTAGTAAAAATTGAACCATTA
GGAGTAGCACCCACCAAGGCAAAGAGAAGAGTGGTGCAGAGAGAAAAAA

GAGCAGTGGGAATAGGAGCTTTGTTCCTTGGGTTCTTGGGAGCAGCAGGA
AGCACTATGGGCGCAGCCTCAATGACGCTGACGGTACAGGCCAGACAATTA
TTGTCTGGTATAGTGCAGCAGCAGAACAATTTGCTGAGGGCTATTGAGGCG
CAACAGCATCTGTTGCAACTCACAGTCTGGGGCATCAAGCAGCTCCAGGC
AAGAATCCTGGCTGTGGAAAGATACCTAAAGGATCAACAGCTCCTGGGGAT
TTGGGGTTGCTCTGGAAAACTCATTTGCACCACTGCTGTGCCTTGGAATGC
TAGTTGGAGTAATAAATCTCTGGAACAGATTGGAATCACACGACCTGGATG
GAGTGGGACAGAGAAATTAACAATTACACAAGCTTAATACACTCCTTAATT
GAAGAATCGCAAAACCAGCAAGAAAAGAATGAACAAGAATTATTGGAATT
AGATAAATGGGCAAGTTTGTGGAATTGGTTTAACATAACAAATTGGCTGTG
GTATATAAAATTATTCATAATGATAGTAGGAGGCTTGGTAGGTTTAAGAATAG
TTTTTGCTGTACTTTCTATAGTGAATAGAGTTAGGCAGGGATATTCACCATTA
TCGTTTCAGACCCACCTCCCAACCCCGAGGGGACCCGACAGGCCCGAAGG
AATAGAAGAAGAAGGTGGAGAGAGAGACAGAGACAGATCCATTCGATTAG
TGAACGGATCTCGACGGTTAACTTTTAAAAGAAAAGGGGGGATTGGGGGG
TACAGTGCAGGGGAAAGAATAGTAGACATAATAGCAACAGACATACAAACT
AAAGAATTACAAAAACAAATTACAAAAATTCAAAATTTTATCGATACTAGTA
AGGATCTGCGATCGCGACATTGATTATTGACTAGTTATTAATAGTAATCAATT
ACGGGGTCATTAGTTCATAGCCCATATATGGAGTTCCGCGTTACATAACTTAC
GGTAAATGGCCCGCCTGGCTGACCGCCCAACGACCCCCGCCCATTGACGTC
AATAATGACGTATGTTCCCATAGTAACGCCAATAGGGACTTTCCATTGACGT
CAATGGGTGGAGTATTTACGGTAAACTGCCCACTTGGCAGTACATCAAGTG
TATCATATGCCAAGTACGCCCCCTATTGACGTCAATGACGGTAAATGGCCCG
CCTGGCATTATGCCCAGTACATGACCTTATGGGACTTTCCTACTTGGCAGTA
CATCTACGTATTAGTCATCGCTATTACCATGGTGATGCGGTTTTGGCAGTACA
TCAATGGGCGTGGATAGCGGTTTGACTCACGGGGATTTCCAAGTCTCCACC
CCATTGACGTCAATGGGAGTTTGTTTTGGCACCAAAATCAACGGGACTTTC
CAAAATGTCGTAACAACTCCGCCCCATTGACGCAAATGGGCGGTAGGCGTG
TACGGTGGGAGGTCTATATAAGCAGTCTAGAGATCCGACGCCGCCATCTCTA
GGCCCGCGCCGGCCCCCTCGCACAGACTTGTGGGAGAAGCTCGGCTACTC
CCCTGCCCCGGTTAATTTGCATATAATATTTCCTAGTAACTATAGAGGCTTAA
TGTGCGATAAAAGACAGATAATCTGTTCTTTTTAATACTAGCTACATTTTACA
TGATAGGCTTGGATTTCTATAAGAGATACAAATACTAAATTATTATTTTAAAA
AACAGCACAAAAGGAAACTCACCCTAACTGTAAAGTAATTGTGTGTTTTGA
GACTATAAATATGCATGCGAGAAAAGCCTTGTTTGCCACCATGGAACGGCT
CGGAGATCATCATTGCGNNNNNNNGTGAGCAAGGGCGAGGAGCTGTTCAC
CGGGGTGGTGCCCATCCTGGTCGAGCTGGACGGCGACGTAAACGGCCACA
AGTTCAGCGTGTCCGGCGAGGGCGAGGGCGATGCCACCTACGGCAAGCTG
ACCCTGAAGTTCATCTGCACCACCGGCAAGCTGCCCGTGCCCTGGCCCACC
CTCGTGACCACCCTGACCTACGGCGTGCAGTGCTTCAGCCGCTACCCCGAC
CACATGAAGCAGCACGACTTCTTCAAGTCCGCCATGCCCGAAGGCTACGTC
CAGGAGCGCACCATCTTCTTCAAGGACGACGGCAACTACAAGACCCGCGC
CGAGGTGAAGTTCGAGGGCGACACCCTGGTGAACCGCATCGAGCTGAAGG
GCATCGACTTCAAGGAGGACGGCAACATCCTGGGGCACAAGCTGGAGTAC

AACTACAACAGCCACAACGTCTATATCATGGCCGACAAGCAGAAGAACGG
CATCAAGGTGAACTTCAAGATCCGCCACAACATCGAGGACGGCAGCGTGC
AGCTCGCCGACCACTACCAGCAGAACACCCCCATCGGCGACGGCCCCGTG
CTGCTGCCCGACAACCACTACCTGAGCACCCAGTCCAAGCTGAGCAAAGA
CCCCAACGAGAAGCGCGATCACATGGTCCTGCTGGAGTTCGTGACCGCCG
CCGGGATCACTCTCGGCATGGACGAGCTGTACAAGGGATCCGCGGCCGCT
GAGGGCAGAGGAAGTCTTCTAACATGCGGTGACGTGGAGGAGAATCCCGG
CCCTTCCGGGATGACCGAGTACAAGCCCACGGTGCGCCTCGCCACCCGCG
ACGACGTCCCCAGGGCCGTACGCACCCTCGCCGCCGCGTTCGCCGACTAC
CCCGCCACGCGCCACACCGTCGATCCGGACCGCCACATCGAGCGGGTCAC
CGAGCTGCAAGAACTCTTCCTCACGCGCGTCGGGCTCGACATCGGCAAGG
TGTGGGTCGCGGACGACGGCGCCGCGGTGGCGGTCTGGACCACGCCGGA
GAGCGTCGAAGCGGGGGCGGTGTTCGCCGAGATCGGCCCGCGCATGGCCG
AGTTGAGCGGTTCCCGGCTGGCCGCGCAGCAACAGATGGAAGGCCTCCTG
GCGCCGCACCGGCCCAAGGAGCCCGCGTGGTTCCTGGCCACCGTCGGCGT
CTCGCCCGACCACCAGGGCAAGGGTCTGGGCAGCGCCGTCGTGCTCCCCG
GAGTGGAGGCGGCCGAGCGCGCCGGGGTGCCCGCCTTCCTGGAGACCTCC
GCGCCCCGCAACCTCCCCTTCTACGAGCGGCTCGGCTTCACCGTCACCGCC
GACGTCGAGGTGCCCGAAGGACCGCGCACCTGGTGCATGACCCGCAAGCC
CGGTGCCTGAATCTAGGTCGACAATCAACCTCTGGATTACAAAATTTGTGA
AAGATTGACTGGTATTCTTAACTATGTTGCTCCTTTTACGCTATGTGGATACG
CTGCTTTAATGCCTTTGTATCATGCTATTGCTTCCCGTATGGCTTTCATTTTCT
CCTCCTTGTATAAATCCTGGTTGCTGTCTCTTTATGAGGAGTTGTGGCCCGT
TGTCAGGCAACGTGGCGTGGTGTGCACTGTGTTTGCTGACGCAACCCCCA
CTGGTTGGGGCATTGCCACCACCTGTCAGCTCCTTTCCGGGACTTTCGCTT
TCCCCCTCCCTATTGCCACGGCGGAACTCATCGCCGCCTGCCTTGCCCGCT
GCTGGACAGGGGCTCGGCTGTTGGGCACTGACAATTCCGTGGTGTTGTCG
GGGAAATCATCGTCCTTTCCTTGGCTGCTCGCCTGTGTTGCCACCTGGATTC
TGCGCGGGACGTCCTTCTGCTACGTCCCTTCGGCCCTCAATCCAGCGGACC
TTCCTTCCCGCGGCCTGCTGCCGGCTCTGCGGCCTCTTCCGCGTCTTCGCCT
TCGCCCTCAGACGAGTCGGATCTCCCTTTGGGCCGCCTCCCCGCCTGGTAC
CTTTAAGACCAATGACTTACAAGGCAGCTGTAGATCTTAGCCACTTTTTAAA
AGAAAAGGGGGGACTGGAAGGGCTAATTCACTCCCAACGAAAATAAGATC
TGCTTTTTGCTTGTACTGGGTCTCTCTGGTTAGACCAGATCTGAGCCTGGGA
GCTCTCTGGCTAACTAGGGAACCCACTGCTTAAGCCTCAATAAAGCTTGCC
TTGAGTGCTTCAAGTAGTGTGTGCCCGTCTGTTGTGTGACTCTGGTAACTA
GAGATCCCTCAGACCCTTTTAGTCAGTGTGGAAAATCTCTAGCAGTAGTAG
TTCATGTCATCTTATTATTCAGTATTTATAACTTGCAAAGAAATGAATATCAG
AGAGTGAGAGGAACTTGTTTATTGCAGCTTATAATGGTTACAAATAAAGCA
ATAGCATCACAAATTTCACAAATAAAGCATTTTTTTCACTGCATTCTAGTTGT
GGTTTGTCCAAACTCATCAATGTATCTTATCATGTCTGGCTCTAGCTATCCCG
CCCCTAACTCCGCCCAGTTCCGCCCATTCTCCGCCCCATGGCTGACTAATTT
TTTTTATTTATGCAGAGGCCGAGGCCGCCTCGGCCTCTGAGCTATTCCAGAA
GTAGTGAGGAGGCTTTTTTGGAGGCCTAGACTTTTGCAGAGACGGCCCAA

ATTCGTAATCATGGTCATAGCTGTTTCCTGTGTGAAATTGTTATCCGCTCACA
ATTCCACACAACATACGAGCCGGAAGCATAAAGTGTAAAGCCTGGGGTGC
CTAATGAGTGAGCTAACTCACATTAATTGCGTTGCGCTCACTGCCCGCTTTC
CAGTCGGGAAACCTGTCGTGCCAGCTGCATTAATGAATCGGCCAACGCGCG
GGGAGAGGCGGTTTGCGTATTGGGCGCTCTTCCGCTTCCTCGCTCACTGAC
TCGCTGCGCTCGGTCGTTCGGCTGCGGCGAGCGGTATCAGCTCACTCAAAG
GCGGTAATACGGTTATCCACAGAATCAGGGGATAACGCAGGAAAGAACATG
TGAGCAAAAGGCCAGCAAAAGGCCAGGAACCGTAAAAAGGCCGCGTTGC
TGGCGTTTTTCCATAGGCTCCGCCCCCCTGACGAGCATCACAAAAATCGAC
GCTCAAGTCAGAGGTGGCGAAACCCGACAGGACTATAAAGATACCAGGCG
TTTCCCCCTGGAAGCTCCCTCGTGCGCTCTCCTGTTCCGACCCTGCCGCTTA
CCGGATACCTGTCCGCCTTTCTCCCTTCGGGAAGCGTGGCGCTTTCTCATAG
CTCACGCTGTAGGTATCTCAGTTCGGTGTAGGTCGTTCGCTCCAAGCTGGG
CTGTGTGCACGAACCCCCCGTTCAGCCCGACCGCTGCGCCTTATCCGGTAA
CTATCGTCTTGAGTCCAACCCGGTAAGACACGACTTATCGCCACTGGCAGC
AGCCACTGGTAACAGGATTAGCAGAGCGAGGTATGTAGGCGGTGCTACAG
AGTTCTTGAAGTGGTGGCCTAACTACGGCTACACTAGAAGGACAGTATTTG
GTATCTGCGCTCTGCTGAAGCCAGTTACCTTCGGAAAAAGAGTTGGTAGCT
CTTGATCCGGCAAACAAACCACCGCTGGTAGCGGTGGTTTTTTTGTTTGCA
AGCAGCAGATTACGCGCAGAAAAAAAGGATCTCAAGAAGATCCTTTGATC
TTTTCTACGGGGTCTGACGCTCAGTGGAACGAAAACTCACGTTAAGGGATT
TTGGTCATGAGATTATCAAAAAGGATCTTCACCTAGATCCTTTTAAATTAAA
AATGAAGTTTTAAATCAATCTAAAGTATATATGAGTAAACTTGGTCTGACAG
TTACCAATGCTTAATCAGTGAGGCACCTATCTCAGCGATCTGTCTATTTCGTT
CATCCATAGTTGCCTGACTCCCCGTCGTGTAGATAACTACGATACGGGAGGG
CTTACCATCTGGCCCCAGTGCTGCAATGATACCGCGAGACCCACGCTCACC
GGCTCCAGATTTATCAGCAATAAACCAGCCAGCCGGAAGGGCCGAGCGCA
GAAGTGGTCCTGCAACTTTATCCGCCTCCATCCAGTCTATTAATTGTTGCCG
GGAAGCTAGAGTAAGTAGTTCGCCAGTTAATAGTTTGCGCAACGTTGTTGC
CATTGCTACAGGCATCGTGGTGTCACGCTCGTCGTTTGGTATGGCTTCATTC
AGCTCCGGTTCCCAACGATCAAGGCGAGTTACATGATCCCCCATGTTGTGC
AAAAAAGCGGTTAGCTCCTTCGGTCCTCCGATCGTTGTCAGAAGTAAGTTG
GCCGCAGTGTTATCACTCATGGTTATGGCAGCACTGCATAATTCTCTTACTG
TCATGCCATCCGTAAGATGCTTTTCTGTGACTGGTGAGTACTCAACCAAGTC
ATTCTGAGAATAGTGTATGCGGCGACCGAGTTGCTCTTGCCCGGCGTCAAT
ACGGGATAATACCGCGCCACATAGCAGAACTTTAAAAGTGCTCATCATTGG
AAAACGTTCTTCGGGGCGAAAACTCTCAAGGATCTTACCGCTGTTGAGATC
CAGTTCGATGTAACCCACTCGTGCACCCAACTGATCTTCAGCATCTTTTACT
TTCACCAGCGTTTCTGGGTGAGCAAAAACAGGAAGGCAAAATGCCGCAAA
AAAGGGAATAAGGGCGACACGGAAATGTTGAATACTCATACTCTTCCTTTT
TCAATATTATTGAAGCATTTATCAGGGTTATTGTCTCATGAGCGGATACATAT
TTGAATGTATTTAGAAAAATAAACAAATAGGGGTTCCGCGCACATTTCCCCG
AAAAGTGCCACCTGACGTCTAAGAAACCATTATTATCATGACATTAACCTAT
AAAAATAGGCGTATCACGAGGCCCTTTCGTCTCGCGCGTTTCGGTGATGAC

GGTGAAAACCTCTGACACATGCAGCTCCCGGAGACGGTCACAGCTTGTCT
GTAAGCGGATGCCGGGAGCAGACAAGCCCGTCAGGGCGCGTCAGCGGGT
GTTGGCGGGTGTCGGGGCTGGCTTAACTATGCGGCATCAGAGCAGATTGTA
CTGAGAGTGCACCATATGCGGTGTGAAATACCGCACAGATGCGTAAGGAGA
AAATACCGCATCAGGCGCCATTCGCCATTCAGGCTGCGCAACTGTTGGGAA
GGGCGATCGGTGCGGGCCTCTTCGCTATTACGCCAGCTGGCGAAAGGGGG
ATGTGCTGCAAGGCGATTAAGTTGGGTAACGCCAGGGTTTTCCCAGTCACG
ACGTTGTAAAACGACGGCCAGTGCCAAGCTG

Supplementary Figure 5. Sequence of GFP reporter construct for PAM screening. CMV promoter was shown in blue; target sequence was shown in red; GFP sequence was shown in green.

**Supplementary information,** **Table S1. Primers and gRNA used in this study**		
**Name**	**Primer sequence**	**Description**
PAM library	gcgagaaaagccttgtttgccaccATGGAACGGCTCGGAGATCATCATTGCGNNNNNNNNgtgagcaagggcgagga	Oligonucleotide for construction of PAM library
AAVS1--1-F	ccg GTAGAGGCGGCCACGACCTG	Oligonucleotide pairs for construction of gRNA expression plasmid on epiCRISPR vector
AAVS1--1-R	aac CAGGTCGTGGCCGCCTCTAC	Oligonucleotide pairs for construction of gRNA expression plasmid on epiCRISPR vector
AAVS1--2-F	ccg GTGAGAATGGTGCGTCCTAG	Oligonucleotide pairs for construction of gRNA expression plasmid on epiCRISPR vector
AAVS1--2-R	aac CTAGGACGCACCATTCTCAC	Oligonucleotide pairs for construction of gRNA expression plasmid on epiCRISPR vector
COMMD2--1-F	ccg GAAAGTCAGAAGGCTGTTGA	Oligonucleotide pairs for construction of gRNA expression plasmid on epiCRISPR vector
COMMD2--1-R	aac TCAACAGCCTTCTGACTTTC	Oligonucleotide pairs for construction of gRNA expression plasmid on epiCRISPR vector
COMMD2--2-F	ccg GCCTTCGTAGATTTTTGGGT	Oligonucleotide pairs for construction of gRNA expression plasmid on epiCRISPR vector
COMMD2--2-R	aac ACCCAAAAATCTACGAAGGC	Oligonucleotide pairs for construction of gRNA expression plasmid on epiCRISPR vector

COMMD2--3-F	ccg CGTGGGGGCGGTGTTCTGTG	Oligonucleotide pairs for construction of gRNA expression plasmid on epiCRISPR vector
COMMD2--3-R	aac CACAGAACACCGCCCCCACG	Oligonucleotide pairs for construction of gRNA expression plasmid on epiCRISPR vector
COMMD2--4-F	ccg CAGAAGGCTGTTGAGTGCCA	Oligonucleotide pairs for construction of gRNA expression plasmid on epiCRISPR vector
COMMD2--4-R	aac TGGCACTCAACAGCCTTCTG	Oligonucleotide pairs for construction of gRNA expression plasmid on epiCRISPR vector
COMMD2--5-F	ccg GGAAAGGGGAAGGCTCCGC A	Oligonucleotide pairs for construction of gRNA expression plasmid on epiCRISPR vector
COMMD2--5-R	aac CGGCAGTCCGTCGTACCCAT	Oligonucleotide pairs for construction of gRNA expression plasmid on epiCRISPR vector
COMMD2--6-F	ccg GCTCCGCAGGCTTCTAGGGC	Oligonucleotide pairs for construction of gRNA expression plasmid on epiCRISPR vector
COMMD2--6-R	aac GCCCTAGAAGCCTGCGGAGC	Oligonucleotide pairs for construction of gRNA expression plasmid on epiCRISPR vector
HGH1--1-F	ccg GACTGAGGTTGGCTAGCGCG	Oligonucleotide pairs for construction of gRNA expression plasmid on epiCRISPR vector
HGH1--1-R	aac CGCGCTAGCCAACCTCAGTC	Oligonucleotide pairs for construction of gRNA expression plasmid on epiCRISPR vector
HGH1--2-F	ccg CGTGTGCAGCGCTCATGGCG	Oligonucleotide pairs for construction of gRNA expression plasmid on epiCRISPR vector
HGH1--2-R	aac CGCCATGAGCGCTGCACACG	Oligonucleotide pairs for construction of gRNA expression plasmid on epiCRISPR vector
HGH1--3-F	ccg GGTAGTGCAGGGGCGCGCGG	Oligonucleotide pairs for construction of gRNA expression plasmid on epiCRISPR vector
HGH1--3-R	aac CCGCGCGCCCCTGCACTACC	Oligonucleotide pairs for construction of gRNA expression plasmid on epiCRISPR vector

HGH1--4-F	ccg CAGGCGCGCTGGCAGCCCGG	Oligonucleotide pairs for construction of gRNA expression plasmid on epiCRISPR vector
HGH1--4-R	aac CCGGGCTGCCAGCGCGCCTG	Oligonucleotide pairs for construction of gRNA expression plasmid on epiCRISPR vector
HGH1--5-F	ccg GACTCGGGCCTGGAGCGGCT	Oligonucleotide pairs for construction of gRNA expression plasmid on epiCRISPR vector
HGH1--5-R	aac AGCCGCTCCAGGCCCGAGTC	Oligonucleotide pairs for construction of gRNA expression plasmid on epiCRISPR vector
HGH1--6-F	ccg CACACGGCGCCGGCTCGCGA	Oligonucleotide pairs for construction of gRNA expression plasmid on epiCRISPR vector
HGH1--6-R	aac TCGCGAGCCGGCGCCGTGTG	Oligonucleotide pairs for construction of gRNA expression plasmid on epiCRISPR vector
HGH1--7-F	ccg CGGAGCCGGCAGACTCGGGC	Oligonucleotide pairs for construction of gRNA expression plasmid on epiCRISPR vector
HGH1--7-R	aac GCCCGAGTCTGCCGGCTCCG	Oligonucleotide pairs for construction of gRNA expression plasmid on epiCRISPR vector
HGH1--8-F	ccg TGGAGCGGCTGGTGCGCGCG	Oligonucleotide pairs for construction of gRNA expression plasmid on epiCRISPR vector
HGH1--8-R	aac CGCGCGCACCAGCCGCTCCA	Oligonucleotide pairs for construction of gRNA expression plasmid on epiCRISPR vector
FGG--1-F	ccg GAAGCACAGTGCCAGGAACC	Oligonucleotide pairs for construction of gRNA expression plasmid on epiCRISPR vector
FGG--1-R	aac GGTTCCTGGCACTGTGCTTC	Oligonucleotide pairs for construction of gRNA expression plasmid on epiCRISPR vector
FGG--2-F	ccg TTCCCAGTGATATCATGGAT	Oligonucleotide pairs for construction of gRNA expression plasmid on epiCRISPR vector
FGG--2-R	aac ATCCATGATATCACTGGGAA	Oligonucleotide pairs for construction of gRNA expression plasmid on epiCRISPR vector

FGG--3-F	ccg ATCACTGGGAAAGGTAACTG	Oligonucleotide pairs for construction of gRNA expression plasmid on epiCRISPR vector
FGG--3-R	aac CAGTTACCTTTCCCAGTGAT	Oligonucleotide pairs for construction of gRNA expression plasmid on epiCRISPR vector
FGG--4-F	ccg AGTAATGTAAAGGAGAAAGT	Oligonucleotide pairs for construction of gRNA expression plasmid on epiCRISPR vector
FGG--4-R	aac ACTTTCTCCTTTACATTACT	Oligonucleotide pairs for construction of gRNA expression plasmid on epiCRISPR vector
PARP12--1-F	ccg TGAAGGCAAGAAACTGCGTG	Oligonucleotide pairs for construction of gRNA expression plasmid on epiCRISPR vector
PARP12--1-R	aac CACGCAGTTTCTTGCCTTCA	Oligonucleotide pairs for construction of gRNA expression plasmid on epiCRISPR vector
EMX1-sg-F	cacc Gagtccgagcagaagaagaa	Oligonucleotide pairs for construction of gRNA expression plasmid on PX459 vector
EMX1-sg-R	aaac TTCTTCTTCTGCTCGGACTC	Oligonucleotide pairs for construction of gRNA expression plasmid on PX459 vector
VEGFA-sg-F	cacc gggtgggggggagtttgctcc	Oligonucleotide pairs for construction of gRNA expression plasmid on PX459 vector
VEGFA-sg-R	aaac GGAGCAAACTCCCCCCACCC	Oligonucleotide pairs for construction of gRNA expression plasmid on PX459 vector
AAVS1-deep-F	ACACTCTTTCCCTACACGACGCTCTTCCGATCTNNNNgctctgggcggaggaatatg	Primers for amplification of the endogenous targets (The frist-step PCR)
AAVS1-deep-R	ACTGGAGTTCAGACGTGTGCTCTTCCGATCTNNNNtccgtgcgtcagttttacct	Primers for amplification of the endogenous targets (The frist-step PCR)
COMMD2-deep-F	ACACTCTTTCCCTACACGACGCTCTTCCGATCTNNNNcttcctgcctcaagtggaca	Primers for amplification of the endogenous targets (The frist-step PCR)
COMMD2-deep-R	ACTGGAGTTCAGACGTGTGCTCTTCCGATCTNNNNaacaagaccctaggactcgc	Primers for amplification of the endogenous targets (The frist-step PCR)
HGH1-deep-F	ACACTCTTTCCCTACACGAC	Primers for amplification of the

	GCTCTTCCGATCTNNNNggcct gcacgagacattg	endogenous targets (The frist-step PCR)
HGH1-deep-R	ACTGGAGTTCAGACGTGTGC TCTTCCGATCTNNNNttggctgag gttggagagc	Primers for amplification of the endogenous targets (The frist-step PCR)
FGG-deep-F	ACACTCTTTCCCTACACGAC GCTCTTCCGATCTNNNNagattg ttaacctgaaagagaaggt	Primers for amplification of the endogenous targets (The frist-step PCR)
FGG-deep-R	ACTGGAGTTCAGACGTGTGC TCTTCCGATCTNNNNgacttaatg ggtagccactttct	Primers for amplification of the endogenous targets (The frist-step PCR)
PARP12-deep-F	ACACTCTTTCCCTACACGAC GCTCTTCCGATCTNNNNgagca cctctgaaatgtggc	Primers for amplification of the endogenous targets (The frist-step PCR)
PARP12-deep-R	ACTGGAGTTCAGACGTGTGC TCTTCCGATCTNNNNcagagcca acacaggcag	Primers for amplification of the endogenous targets (The frist-step PCR)
Deep-F1	ACACTCTTTCCCTACACGAC GCTCTTCCGATCTNNNN gcgagaaaagccttgttt	Primers for amplification of the endogenous targets (The frist-step PCR)
Deep-R1	ACTGGAGTTCAGACGTGTGC TCTTCCGATCTNNNN ctgaacttgtggccgtttac	Primers for amplification of the endogenous targets (The frist-step PCR)
P5-adapter0-F	AATGATACGGCGACCACCGA GATCTACAC ACATCG ACACTCTTTCCCTACACGAC	Primers for amplification of the endogenous targets (The second-step PCR)
P7-adapter3-R	CAAGCAGAAGACGGCATACG AGAT CACTGT GTGACTGGAGTTCAGACGTG TG	Primers for amplification of the endogenous targets (The second -step PCR)
P7-adapter4-R	CAAGCAGAAGACGGCATACG AGAT ATTGGC GTGACTGGAGTTCAGACGTG TG	Primers for amplification of the endogenous targets (The second -step PCR)
P7-adapter5-R	CAAGCAGAAGACGGCATACG AGAT GATCTG GTGACTGGAGTTCAGACGTG TG	Primers for amplification of the endogenous targets (The second -step PCR)
P7-adapter6-R	CAAGCAGAAGACGGCATACG AGAT TACAAG GTGACTGGAGTTCAGACGTG TG	Primers for amplification of the endogenous targets (The second -step PCR)

P7-adapter7-R	CAAGCAGAAGACGGCATACG AGAT CGTGAT GTGACTGGAGTTCAGACGTG TG	Primers for amplification of the endogenous targets (The second -step PCR)
P7-adapter8-R	CAAGCAGAAGACGGCATACG AGAT GCCTAA GTGACTGGAGTTCAGACGTG TG	Primers for amplification of the endogenous targets (The second -step PCR)
P7-adapter9-R	CAAGCAGAAGACGGCATACG AGAT TCAAGT GTGACTGGAGTTCAGACGTG TG	Primers for amplification of the endogenous targets (The second -step PCR)
P7-adapter10-R	CAAGCAGAAGACGGCATACG AGAT CTGATC GTGACTGGAGTTCAGACGTG TG	Primers for amplification of the endogenous targets (The second -step PCR)
P7-adapter11-R	CAAGCAGAAGACGGCATACG AGAT AAGCTA GTGACTGGAGTTCAGACGTG TG	Primers for amplification of the endogenous targets (The second -step PCR)
P7-adapter12-R	CAAGCAGAAGACGGCATACG AGAT GTAGCC GTGACTGGAGTTCAGACGTG TG	Primers for amplification of the endogenous targets (The second -step PCR)
P7-adapter13-R	CAAGCAGAAGACGGCATACG AGAT TTGACT GTGACTGGAGTTCAGACGTG TG	Primers for amplification of the endogenous targets (The second -step PCR)
P7-adapter14-R	CAAGCAGAAGACGGCATACG AGAT GGAACT GTGACTGGAGTTCAGACGTG TG	Primers for amplification of the endogenous targets (The second -step PCR)
P7-adapter15-R	CAAGCAGAAGACGGCATACG AGAT TGACAT GTGACTGGAGTTCAGACGTG TG	Primers for amplification of the endogenous targets (The second -step PCR)
P7-adapter16-R	CAAGCAGAAGACGGCATACG AGAT GGACGG GTGACTGGAGTTCAGACGTG TG	Primers for amplification of the endogenous targets (The second -step PCR)
P7-adapter17-R	CAAGCAGAAGACGGCATACG AGAT CTCTAC GTGACTGGAGTTCAGACGTG	Primers for amplification of the endogenous targets (The second -step PCR)

	TG	
P7-adapter18-R	CAAGCAGAAGACGGCATACG AGAT GCGGAC GTGACTGGAGTTCAGACGTG TG	Primers for amplification of the endogenous targets (The second -step PCR)
P7-adapter19-R	CAAGCAGAAGACGGCATACG AGAT TTTCAC GTGACTGGAGTTCAGACGTG TG	Primers for amplification of the endogenous targets (The second -step PCR)
P7-adapter20-R	CAAGCAGAAGACGGCATACG AGAT GGCCAC GTGACTGGAGTTCAGACGTG TG	Primers for amplification of the endogenous targets (The second -step PCR)
P7-adapter21-R	CAAGCAGAAGACGGCATACG AGAT CGAAAC GTGACTGGAGTTCAGACGTG TG	Primers for amplification of the endogenous targets (The second -step PCR)
P7-adapter22-R	CAAGCAGAAGACGGCATACG AGAT CGTACG GTGACTGGAGTTCAGACGTG TG	Primers for amplification of the endogenous targets (The second -step PCR)
P7-adapter23-R	CAAGCAGAAGACGGCATACG AGAT CCACTC GTGACTGGAGTTCAGACGTG TG	Primers for amplification of the endogenous targets (The second -step PCR)
P7-adapter24-R	CAAGCAGAAGACGGCATACG AGAT GCTACC GTGACTGGAGTTCAGACGTG TG	Primers for amplification of the endogenous targets (The second -step PCR)
Deep-F1	ACACTCTTTCCCTACACGAC GCTCTTCCGATCTNNNNN gcgagaaaagccttgttt	Primers to amplify the random PAM for deep sequencing
Deep-R1	ACTGGAGTTCAGACGTGTGC TCTTCCGATCTNNNNN ctgaacttgtggccgtttac	Primers to amplify the random PAM for deep sequencing
P5-adapter0-F	AATGATACGGCGACCACCGA GATCTACAC ACATCG ACACTCTTTCCCTACACGAC	Primers to amplify the random PAM for deep sequencing
P7-adapter3-R	CAAGCAGAAGACGGCATACG AGAT CACTGT GTGACTGGAGTTCAGACGTG TG	Primers to amplify the random PAM for deep sequencing
EMX1-OT5-F	AagtccgagGagaGgaagaaAGGgtga gcaagggcgaggag	Primers for plasmid construction

EMX1-OT9-F	GagtAcAagcagaTgaaAaaCGGgtg agcaagggcgaggag	Primers for plasmid construction
EMX1-OT13-F	GagGccAagcagaaAGaAaaAGGgt gagcaagggcgaggag	Primers for plasmid construction
EMX1-OT31-F	GaCtccgagcagCagaagGaTGGgtga gcaagggcgaggag	Primers for plasmid construction
EMX1-OT36-F	GagtTAgagcagaGgaagaGAGGgtg agcaagggcgaggag	Primers for plasmid construction
EMX1-OT52-F	GTgtcAgagcagaaAaagaGTGGgtg agcaagggcgaggag	Primers for plasmid construction
VEGFA-OT5-F	gggGgCAgggagAttgctccTGGgtga gcaagggcgaggag	Primers for plasmid construction
VEGFA-OT7-F	GTAAgtAAgggAagtttgctccTGGgt gagcaagggcgaggag	Primers for plasmid construction
VEGFA-OT19-F	gggAggAgAgagtttgctcTCTGgtgag caagggcgaggag	Primers for plasmid construction
VEGFA-OT33-F	AgAggggTggagtttgTtccAGGgtga gcaagggcgaggag	Primers for plasmid construction
VEGFA-OT49-F	gggGAgggggagAtGgctccCGGgtg agcaagggcgaggag	Primers for plasmid construction
VEGFA-OT60-F	GAGAggtggggTgatttgctccAGGgt gagcaagggcgaggag	Primers for plasmid construction
cozak-R	CATggtggcaaacaaggctt	Primers for plasmid construction
**Name**	**gRNA sequence**	**Description**
AAVS1--sg1	GTAGAGGCGGCCACGACCTG	gRNA for off-target decetion of SpCas9 in endogenous site of human
AAVS1--sg2	GTGAGAATGGTGCGTCCTAG	gRNA for off-target decetion of SpCas9 in endogenous site of human
COMMD2--sg1	GAAAGTCAGAAGGCTGTTGA	gRNA for off-target decetion of SpCas9 in endogenous site of human
COMMD2--sg2	GCCTTCGTAGATTTTTGGGT	gRNA for off-target decetion of SpCas9 in endogenous site of human
COMMD2--sg3	CGTGGGGGCGGTGTTCTGTG	gRNA for off-target decetion of SpCas9 in endogenous site of human
COMMD2--sg4	CAGAAGGCTGTTGAGTGCCA	gRNA for off-target decetion of SpCas9 in endogenous site of human

COMMD2--sg5	GGAAAGGGGAAGGCTCCGCA	gRNA for off-target decetion of SpCas9 in endogenous site of human
COMMD2--sg6	GCTCCGCAGGCTTCTAGGGC	gRNA for off-target decetion of SpCas9 in endogenous site of human
HGH1--sg1	GACTGAGGTTGGCTAGCGCG	gRNA for off-target decetion of SpCas9 in endogenous site of human
HGH1--sg2	CGTGTGCAGCGCTCATGGCG	gRNA for off-target decetion of SpCas9 in endogenous site of human
HGH1--sg3	GGTAGTGCAGGGGCGCGCGG	gRNA for off-target decetion of SpCas9 in endogenous site of human
HGH1--sg4	CAGGCGCGCTGGCAGCCCGG	gRNA for off-target decetion of SpCas9 in endogenous site of human
HGH1--sg5	GACTCGGGCCTGGAGCGGCT	gRNA for off-target decetion of SpCas9 in endogenous site of human
HGH1--sg6	CACACGGCGCCGGCTCGCGA	gRNA for off-target decetion of SpCas9 in endogenous site of human
HGH1--sg7	CGGAGCCGGCAGACTCGGGC	gRNA for off-target decetion of SpCas9 in endogenous site of human
HGH1--sg8	TGGAGCGGCTGGTGCGCGCG	gRNA for off-target decetion of SpCas9 in endogenous site of human
FGG--sg1	GAAGCACAGTGCCAGGAACC	gRNA for off-target decetion of SpCas9 in endogenous site of human
FGG--sg2	TTCCCAGTGATATCATGGAT	gRNA for off-target decetion of SpCas9 in endogenous site of human
FGG--sg3	ATCACTGGGAAAGGTAACTG	gRNA for off-target decetion of SpCas9 in endogenous site of human
FGG--sg4	AGTAATGTAAAGGAGAAAGT	gRNA for off-target decetion of SpCas9 in endogenous site of human
PARP12--sg1	TGAAGGCAAGAAACTGCGTG	gRNA for off-target decetion of

		SpCas9 in endogenous site of human
EMX1-sg	gagtccgagcagaagaagaa	gRNA for off-target detection by GFP reporter-based approach
VEGFA-sg	gggtgggggggagtttgctcc	gRNA for off-target detection by GFP reporter-based approach
GFP-qPCR-F	AAGGACGACGGCAACTACAA	The primer pair for qPCR
GFP-qPCR-R	TCTGCTTGTCGGCCATGATA	The primer pair for qPCR
